# Dental Association

**Published:** 1862-11

**Authors:** 


"DENTAL ASSOCIATION."

The following report of a convention of Dentists of Western New York, held in this city, has been handed us for publication:—

A convention of Dentists of Western New York was held at Irving Hall, Rochester, on the first day of October, at which they proceeded to form a permanent organization known by the name of "The Dental Association of Western New York."

The territory embraced within the limits is that portion of the State lying west of Cayuga Lake, including the village of Ithica and the western part of Tompkins and Chemung on the south, and the county of Wayne.

The objects sought to be attained are to "cultivate the science and art of dentistry and all its collateral branches, to elevate and sustain the professional character of dentists, to promote among them mutual improvement, social intercourse and good feeling, and to collectively represent and have cognizance of the common interests of the dental profession in Western New York."

Dr. Chas. W. Harvey, of Buffalo, was elected President, Dr. E. F. Wilson, of Rochester, Vice-President, Dr. L. W. Bristol, of Lockport, Secretary, and Dr. B. T. Whitney, of Buffalo, Treas'r.

"All practicing dentists within the Territory of Western New York, in good standing, shall be eligible to membership, and may become members by signing the articles of the Association and paying an initiation fee.

The Association is to hold two regular meetings in each year, at such place as the last meeting shall designate, the annual meeting to be held on the first Tuesday in October, and the semi-annual on the first Tuesday in May.

A good number of dentists were present and perfected their membership, expressing their determination to follow up the Association so auspiciously commenced, and believed to be of such vital importance to the profession."—*Rochester paper.*

By the above it will be seen that our professional brethren of Western New York are up and doing, in the organization of a Dental Society for that section of the State. This gives us much gratification and pleasure. There is material in that region for a splendid association.

The vitality, development and standing of the profession depends in a large degree upon the forming and sustaining just such organizations throughout the country.

How differently men view each other, when they come into immediate professional association: prejudices and jealousies vanish and flee away, under the benign influences of association, and a mutual desire to help each other and be assisted springs up. Almost every department of human industry recognizes and acts by the mighty power of associated effort. And why not the dental profession avail itself of this potent agency.

The man who stands aloof from association and comparison with his fellows is either ignorant and ashamed to confess his ignorance, (oh, how noble a thing it is to see a man stand up, and with an open, honest, manly inquiring face, say, unhesitatingly, *I don't know*,) or is a pirate upon his profession, for his own niggardly purpose, and selfish aggrandisement. Such a man never says I don't know, but he assumes to know every thing; but against such a man his own face is a swift and sure witness. This is intended to apply only to those upon whom the shoe fits closely. T.

